# CRISPR-Cas system: a potential alternative tool to cope antibiotic resistance

**DOI:** 10.1186/s13756-020-00795-6

**Published:** 2020-08-10

**Authors:** Bilal Aslam, Maria Rasool, Adi Idris, Saima Muzammil, Roman Farooq Alvi, Mohsin Khurshid, Muhammad Hidayat Rasool, Derong Zhang, Zhongren Ma, Zulqarnain Baloch

**Affiliations:** 1 Biomedical Research Center, Northwest Minzu University, Lanzhou, 730030 P. R. China; 2 Department of Microbiology, Government College University Faisalabad, Faisalabad, Pakistan; 3 Menzies Health Institute Queensland and School of Medical Science, Griffith University, Southport, QLD Australia

**Keywords:** Antibiotic resistance, CRISPR-Cas

## Abstract

Antibiotic exposure leads to massive selective pressures that initiate the emergence and spread of antibiotic resistance in commensal and pathogenic bacteria. The slow process of developing new antibiotics makes this approach counterintuitive for combatting the rapid emergence of new antibiotic resistant pathogens. Therefore, alternative approaches such as, the development of nucleic acid-based anti-bacterial treatments, anti-bacterial peptides, bacteriocins, anti-virulence compounds and bacteriophage therapies should be exploited to cope infections caused by resistant superbugs. In this editorial, we discuss how the newly popular CRISPR-Cas system has been applied to combat antibiotic resistance.

## Introduction

The rapid development of resistant superbugs and the arms race between bactericidal agents and multidrug-resistant bacteria is the most burning public health issue across the globe. Formulating novel anti-bacterials to combat resistant infections is the topmost priority in medicine, but a clear solution to this problem has yet to be found. In 2013, according to the centre for disease control (CDC) report, annually around 2 million people have been infected by antibiotic-resistant bacteria, out of which 23,000 people have died [4]. The excessive use and misuse of antibiotics has led to the rapid development of resistance and bacterial adaptation to available treatments. Screening, testing, and formulation of antibiotics are recourse intensive and costly, which limits alternative treatment options against resistant bacterial strains. In 2017, about 41 new antimicrobials were in clinical trials [1] out of which, only 20% have been approved [8]. Since the success rate for the development and approval for new antibiotics is very low, alternative research strategies such as, the development of nucleic acid-based anti-bacterial treatments, anti-bacterial peptides, bacteriocins, anti-virulence compounds and bacteriophage therapies should be exploited to cope infections caused by resistant superbugs [6].

A potential and adaptable method is to implement a revolutionary technology; “the type II CRISPR-Cas system”, a system based on the bacterial adaptive immune system aimed to counter ‘the invasion by foreign genetic material’ such as, bacteriophages as well as mobile genetic elements. CRISPR-Cas9 is an “RNA-guided-DNA cutter”. Upon bacteriophage infection inside the bacteria, the Cas machinery barcodes small phage genome sequences into the genome of bacteria to counter-attack using CRISPR-Cas9 to cleave foreign genetic material [2]. One of the most dynamic and highly specific key features of this system is ‘sequence-specific targeting’, the ability to distinguish between commensal and pathogenic bacterial species. Guide CRISPR-RNA can be constructed to target only chromosomal and virulence genes that are highly specific to pathogens, therefore, enabling this revolutionary system to be reused against the bacteria rather defending against invaders [5]. For instance, the newly developed CRISPR/Cas9 “pro-active” genetic system (Pro-AG) could potentially be used to eliminate of bacterial virulence factors carried on virulence plasmids and resistance determinants in commensal bacteria [9]. [16] Since Cas9 has nuclease activity, it can be programmed with a particular target sequence, enhancing the cytotoxicity of resistant cells [17]. Therefore, a CRISPR-guide RNA can be designed specifically to target resistance or virulence genes, it will induce a break inside the dsDNA of resistant bacteria, reverting them into the antibiotic sensitive ones [5].

Care must be taken while designing the gRNAs as any non-specific targeting associated with Cas9 could lead to collateral killing of commensal bacteria leading to microbiota dysbiosis. Metagenomic studies have taught us that this dysbiosis is association with several diseases in man. This could be potentially adverse in those who are immunocompromised (e.g. cancer patients undergoing chemotherapy or organ transplant patients). Considering these facts, it would be feasible to co-administer CRISPR-Cas-based treatments with probiotics to maintain microflora homeostasis in microflora [12]. Given that bacteria can carry different resistance mechanism for a single drug and often possess many alleles for a gene encoding a single resistance mechanism, gRNA design and selection methodology for target region should therefore be stringent.

For the delivery of CRISPR-Cas9 anti-bacterial package, CRISPR-Cas9 targeting bacterial chromosomal genes can be encapsulated into phage capsids via genetically encoding the machinery in a phagemid, thus ensuring ‘species-specific killing of pathogenic strains while sparing non-targeted ones [3]. This revolutionary strategy may successfully repurpose to attack, rather than defend, the bacteria for treating newly trending resistant infections. Since the majority of resistance genes are found on plasmids, plasmid based CRISPR cas9 system that encodes sgRNA and cas9 protein from the same vector is used to avoid multiple transfections [10]. The delivery challenge increases when the pathogenic bacteria are intracellular. For this, the phage-encoded-Cas9 system should be selectively delivered within the infected host cells, while residing inside the cells before releasing them into the bacteria. In other words, this challenge increases the delivery complexity by adding two layers of specificity; delivery to infected-host cell as well as direct delivery to intracellular bacteria [18]. Alteration of membrane composition by attaching peptide-directing nanoparticles to infected cells or by attaching self-recognition peptides for evading immune clearance could offer potential therapeutic solutions to treat such intracellular microbial infections.

The CRISPR/Cas system can induce various off-target effects, potentially resulting in detrimental outcomes. In saying this, many techniques have been employed to reduce and avoid these off-target effects, and at the same time revamp the on-target efficacy. In many cases, these off target mutations may become better by selecting particular sgRNA with minimal off-targets (based on a potent reference genome sequencing system). Right after the reference genome selection, it is also very important to properly choose a designing tool for sgRNAs and also an effective delivery system with less somaclonal variations. In this regard, many software have been designed/or developed with negligible off-targeting. However, more research is required to enhance on-target efficacy with minimal off-target effects [11].

The phage-encoded Cas9 system is now able to quickly build huge libraries of Cas9-guide RNAs to effectively combat and defeat bacterial resistance. In order to make this revolutionary system as novel antimicrobial agents, PAM sequence specificity, the sequence that guides the Cas9-gRNA complex to the target site, must be characterized which can further be predicted and tested by bioinformatics retrieval and plasmid interference assays [14]. Further advances in phage engineering could also improve this. But the question remains as to how we could adapt our proof of principle lab strain study to emerging and trending biological threats [7]. Although this proposed approach has the potential to particularly kill or re-sensitize the pathogen, the use of this approach in a complex microbial community to eliminate antibiotic resistance genes remains a challenge. Other challenges include; narrow host range of the majority of phage species, resistance evolution via deletions or mutations against this “CRISPR-Cas” system. After formulation of this antimicrobial approach, its efficacy to the matrices for effective therapy and delivery still poses a great challenge. Chemical modification of a drug for delivery reduces the dosage requirement, optimizes the treatment cost and also bypass normal heathy cells [18]. A variety of approaches can be used to edit human host cell including CRISPR-cas9, but these approaches only deals with human host/target cell and are unable to address the challenges associated with the delivery of this system amongst phages to counteract intracellular infections. Furthermore, structural and size variations should also be considered. Due to structural diversity of phages, conventional approaches, like use of nanoparticles, are not effective in cases when phages are non-symmetrical and large. In order to overcome this issue, it is essential to encapsulate bacteriophages to maintain and stabilize them for therapeutic purposes [15].

Future work can overcoming these obstacles related to developing “CRISPR-Cas based antibacterial”. The foremost steps towards the sustainable implementation of this CRISPR- Cas technology includes finding a suitable delivery vector, engineering an appropriate broad host range vector, use of multiplex approach that comprise CRISPR-Cas targeting of various sequences to decrease the resistance prospects [13].

## Conclusion

Taking all of this together, CRISPR-based research has gained a significant amount of attention as the “CRISPR beauty” lies in that it can kill and attack antibiotic-resistant bacteria as easily as an antibiotic sensitive bacterium. Furthermore, this system could potentially only target pathogens while preserving commensal bacteria in the microbiota. The current momentum to predict the ‘future of CRISPR craze’ lies in controlling the composition of the microbial community instead of being used as a conventional broad-spectrum antibiotic.

## Data Availability

N.A

## Abbreviations

CDC: Center for disease control
CRISPR: Clustered regularly interspaced short palindromic repeats
PAM: Protospacer adjacent motif
gRNA: Guide-RNA
